# Recent Developments in Clinical Plasma Proteomics—Applied to Cardiovascular Research

**DOI:** 10.3390/biomedicines10010162

**Published:** 2022-01-12

**Authors:** Nicolai Bjødstrup Palstrøm, Rune Matthiesen, Lars Melholt Rasmussen, Hans Christian Beck

**Affiliations:** 1 Centre for Clinical Proteomics, Department of Clinical Biochemistry and Pharmacology, Odense University Hospital, 5000 Odense, Denmark; Nicolai.Bjodstrup.Palstrom@rsyd.dk (N.B.P.); Lars.Melholt.Rasmussen@rsyd.dk (L.M.R.); 2 Computational and Experimental Biology Group, CEDOC, Chronic Diseases Research Centre, NOVA Medical School, Faculdade de Ciências Médicas, Universidade NOVA de Lisboa, 1169-056 Lisbon, Portugal; Rune.Matthiesen@nms.unl.pt

**Keywords:** mass spectrometry-based proteomics, affinity proteomics, cardiovascular disease, plasma proteomics

## Abstract

The human plasma proteome mirrors the physiological state of the cardiovascular system, a fact that has been used to analyze plasma biomarkers in routine analysis for the diagnosis and monitoring of cardiovascular diseases for decades. These biomarkers address, however, only a very limited subset of cardiovascular diseases, such as acute myocardial infarct or acute deep vein thrombosis, and clinical plasma biomarkers for the diagnosis and stratification cardiovascular diseases that are growing in incidence, such as heart failure and abdominal aortic aneurysm, do not exist and are urgently needed. The discovery of novel biomarkers in plasma has been hindered by the complexity of the human plasma proteome that again transforms into an extreme analytical complexity when it comes to the discovery of novel plasma biomarkers. This complexity is, however, addressed by recent achievements in technologies for analyzing the human plasma proteome, thereby facilitating the possibility for novel biomarker discoveries. The aims of this article is to provide an overview of the recent achievements in technologies for proteomic analysis of the human plasma proteome and their applications in cardiovascular medicine.

## 1. Introduction

In recent years, there has been a rise in the use of proteomic approaches for the study of bodily fluids to uncover disease mechanisms that are associated with the cardiovascular system and to improve diagnosis, prognosis, and monitoring in cardiovascular diseases (CVD). As CVDs remain a prominent cause of morbidity and death, interest in establishing CVD-related biomarkers has steadily grown [1]. Recognizing risk factors such as tobacco usage, diabetes, hypertension, and hyperlipidemia in individuals is currently the dominant method of identifying those who are at risk of developing CVDs. [2]. Currently, some plasma proteins are routinely investigated for decision making in cardiovascular medicine. Examples of the most prominent ones are creatine kinase and troponins, and natriuretic peptides for the diagnosis of myocardial infarcts and heart failure. In addition, lipoproteins are promising for the stratification of cardiovascular risk. However, it is common for these markers that each of them is limited in their diagnostic or predictive accuracy. Therefore, high-performance biomarkers for CVD stratification, diagnosis of acute myocardial infarct (AMI), heart failure (HF), and other cardiovascular conditions, such as abdominal aortic aneurysms are urgently needed. Biomarkers for cardiovascular diagnostics should ideally have a high sensitivity, possess high tissue specificity, and the measured concentration should be proportional to the severity of the specific CVD [3]. The clinical cohorts from which biomarkers for CVD is derived from should be clearly defined in terms of the study population regarding e.g., sex, ethnicity, age, preexisting conditions, and other factors that are known to influence CVDs and also should be accounted for during statistical analysis [4].

The plasma proteome contains thousands of proteins and the general assumption is that most of them remain unexplored for their relation to multiple diseases including CVDs. Therefore, a systematic exploration of all proteins that are present in plasma holds great potential for displaying novel biomarkers for diagnostic, prognostic, and monitoring purposes. Historically, barriers for plasma proteomics have mainly been due to technical limitations, for example, a lack of sensitivity and specificity of the applied analytical method as well as limited possibilities for multiplexing and a low sample throughput. Complementing the information that was obtained from traditional risk factors with measurements of the relevant biomarkers in body fluids, the individualized risk stratification and diagnosis of CVD may lead the way for improved patient classification [5]. Proteomics covers a variety of analysis principles spanning both explorative and unbiased methods. For example, 2-gel electrophoresis (2DE) coupled to matrix-assisted laser desorption time-of-flight mass spectrometry (MALDI-TOF) as well as two-dimension liquid chromatography in combination with tandem mass spectrometry are often applied in discovery proteomics. On the other hand, affinity-based methods such as multiplex immunoassays and aptamer-based assays target arrays of specific proteins [6,7]. In the present paper, we review the highly cited and recent proteomics methods and their application to the plasma proteome in cardiovascular medicine. We discuss the plasma proteome and the analytical challenges in assaying it. The current trends within alleviating analytical limitations are explored along with innovative proteomic technologies that are utilized in cardiovascular research that may improve clinical protein biomarker studies in the future.

## 2. The Human Plasma Proteome and Its Complexity

The human plasma proteome is an immensely complex mixture of proteins. Astonishingly, only 730 proteins are known to be secreted into the blood [8] whereas the vast majority of plasma proteins are present as a result of leakage into the blood due to tissue degradation and damage, i.e., the plasma proteome potentially comprises of proteins that were derived from all tissues. An excellent source for detailed information on the human plasma proteome with lists of proteins that are predicted to be secreted and their origin of expression, as well as a continuously updated list of the human plasma protein concentration of several thousand proteins that are leaked from various tissues into the blood stream are filed by The Human Protein Atlas [9]. This blood protein repository also summarizes lists of plasma proteins that are detected by immunoassays, mass spectrometry, and proximity extension assays [8]. This places plasma as an ideal medium for the clinical analysis of biomarkers that reflect disease states of various diseases including CVDs. A huge variety of proteins, such as complement factors, immunoglobulins, carrier proteins (e.g., albumin), tissue leakage, and messenger proteins (troponins, creatine kinase, natriuretic peptides, and cytokines) with highly diverse functions are, therefore, measurable in human plasma. This enormous range of functions of plasma proteins is accompanied by an extremely dynamic range in the abundance of plasma proteins spanning >11 orders of magnitude [10]. Moreover, relatively few proteins, such as albumin, immunoglobulins, and complement factors, constitute nearly 99% of the total protein concentration of human plasma. Albumin, the largest contributor, covers up to 55% of the total plasma protein concentration [11,12], approximately 70 mg/mL, whereas cytokines and troponins are present in levels below pg/mL. Despite these challenges, plasma is, nevertheless, the most clinically relevant biological fluid for analysis of disease-related proteins in patients. Analyzing the remaining ~1% of the concentration range for disease-related, low-abundant proteins is, therefore, the primary objective of many proteome studies, as these low-abundant circulatory and/or secreted proteins are often regarded as more probable disease-associated candidate biomarkers [13]. Despite decades of research into the human plasma proteome, the exact number of proteins in the human plasma proteome is still unknown. Conservative estimations that are based on the hypothesis that a single gene equals one protein estimates that there are approximately 20,000 proteins in the human proteome [14]. However, this figure does not take into account the presence of different variants of the canonical proteins that are defined in the sequence databases. Different proteoforms of the canonical proteins arise from alternative splicing, single amino acid polymorphisms (SAPs), as well as a large diversity in post-translational processing and modifications (PTMs), which increase the size of the human proteome [15]. PTMs, such as phosphorylation or acetylation’s, can be highly relevant in relation to understanding disease mechanisms. PTMs often regulate function and can be used to gain knowledge about the various aspects of a disease, e.g., pathway activations [16]. Theoretical approximations of the possible number of different human proteoforms are in the range from ~98,000 to several millions, depending on the perspectives that are applied [17,18]. Currently, the experimental validation of proteoforms is hindered by the analytical sensitivity of the existing level of technology in proteomics, since there is unfortunately no way to multiply the copies of single proteins, similar to PCR amplification of nucleic acids beyond the level of detection [14,19]. Specific proteoforms can also be the result of post-translational processing into disease-specific proteins as exemplified by D-dimer for the diagnosis of deep vein thrombosis (DVT) or glycohemoglobin for the diagnosis and monitoring of diabetes, a major risk factor of CVDs.

## 3. Cardiovascular Diseases and Related Biomarkers

Clinical proteome studies for biomarker discovery are reliant on the collection of appropriate samples from both the patients and the controls. Principle proteome analysis can target any kind of fluid or tissue. In practice in cardiovascular research, the samples are most often blood-based biofluids (plasma or serum) or urine, as well as tissue samples from vascular and heart biopsies [20]. The systematic analysis of the plasma proteome provides opportunities for the discovery of novel biomarkers, which can also deepen the pathophysiological understanding of various cardiovascular diseases. Different biomarkers may carry out several diverse functions in a clinical context such as diagnostic, prognostic, predictive, and therapeutic [21]. Diagnostic biomarkers tend to be in high demand because they enable the early detection of diseases, allowing therapeutic options to be given earlier, thus increasing the effectiveness of treatment [22]. Prognostic biomarkers may provide an indication of the likely outcome of the disease irrespective of the treatment. On the other hand, predictive biomarkers are used for identifying patient subpopulations, which would benefit from more tailored therapies due to a larger effect of treatment compared to other patient populations [23]. Therapeutic biomarkers are typically proteins that have been either proven to be directly or indirectly involved in disease progression and are, therefore, an optimal target for the development of therapies [22]. Most plasma biomarker discovery studies aim at investigating circulating biomarkers in plasma as it is a convenient source for sampling requiring little time and effort compared to site-specific tissue samples [24]. The convenience comes at the cost of diminished specificity, as the biomarker in question may represent the disease state of any organ in the body unless it is organ- or disease-specific. It is recognized that while tissue-based proteome analysis is a more direct analysis of proteins from the site of disease and often considered the gold standard for diagnostic and prognostic applications, plasma is far more superior in terms of reproducibility of measurements as well as in terms of non-invasiveness. This type of sampling is more feasible and superior and is preferred over invasive tissue biopsies, which is also prone to heterogeneity in tissue sampling [25,26]. Blood coagulation is prevented by the addition of an anti-coagulant to the sample vial, such as ethylenediaminetetraacetic acid (EDTA) or heparin, prior to extraction. The proximity of plasma to all tissues makes it ideal for protein biomarker discovery as the plasma proteome profile is representative of the general state of the entire organism [27]. The low-risk, minimally invasive method of sample collection makes sequential sampling of research subjects manageable for both the researchers and the participants in larger cohorts. Protein degradation in long-term stored plasma samples is minimal, as previously shown by others, making it ideal for storage in biobanks [28]. Moreover, several pre-analytical variables that are related to venous blood sample collection, management, and storage can negatively influence the quality of the sample, which could result in unwanted variability as well as hindering the comparison across study groups in clinical trials (Table 1) [4,29]. Aside from the risk of incorrect diagnosis and increased cost, the loss of samples due to incorrect sample collection will also impact the statistical power of a potential study [30]. The priority of biobank facilities and clinical laboratories should, therefore, be to implement standard operating procedures in an effort to maintain the integrity of the blood samples, such as the H3-A6 guideline that was issued by the Clinical Laboratory Standards Institute (CLSI) [31].

Many of the major clinically relevant biomarkers for cardiovascular diseases that are in use today were discovered in 1960–1990 and the development of sensitive assays for their measurements have since then been essential in patient care worldwide [32]. Some of the most sensitive biomarkers for myocardial injury are the cardiac troponins I and T (cTnI and cTnT), which are the preferred biomarkers for diagnosing acute myocardial infarcts (AMI) as well as predicting both reinfarction and mortality [33]. Another important biomarker is the B-type natriuretic peptide (BNP) and the N-terminal prohormone (NTproBNP) which have proven useful as a diagnostic tool for heart failure [34]. D-dimer has commonly been used to diagnose acute deep vein thrombosis and pulmonary embolism in emergency departments with high sensitivity [35]. There are, nonetheless, a number of CVDs for which clinically useful biomarkers have not yet been discovered, including abdominal aortic aneurysm (AAA) and cardiac conditions, such as diastolic dysfunction.

## 4. Recent Developments in Assaying the Human Plasma Proteome by Proteomics Methods

As described above, the human plasma proteome displays an extreme complexity in terms of function and protein abundance for specific proteins. This complexity transforms into an extreme analytical complexity when it comes to the proteomic analysis of individual proteins at low abundance in the presence of very high abundant proteins. The predominance of high abundant so-called classical plasma proteins complicates the detection of the lower abundant ones by compromising the dynamic range of the analytical methods in question; in particular, the traditionally used methods for plasma proteome analysis, such gel-based electrophoretic methods (1- and 2-DE) and LC methods combined with MALDI-TOF-MS and MSMS analysis. These methods, introduced in the late 1990s and early 2000s, were limited to the detection of most abundant proteins in plasma. Since then several attempts to counteract the challenges of analyzing low-abundant proteins in plasma have been investigated throughout the years by the introduction of a tremendous number novel methods for sample preparation of human plasma, data acquisition methods for mass spectrometry-based plasma proteomics, multiplex affinity-based assays that rely on binding of specific proteins to short oligomers, or to antibodies enabling the simultaneous targeted measurement of 100 to thousands of proteins.

**Figure 1:**
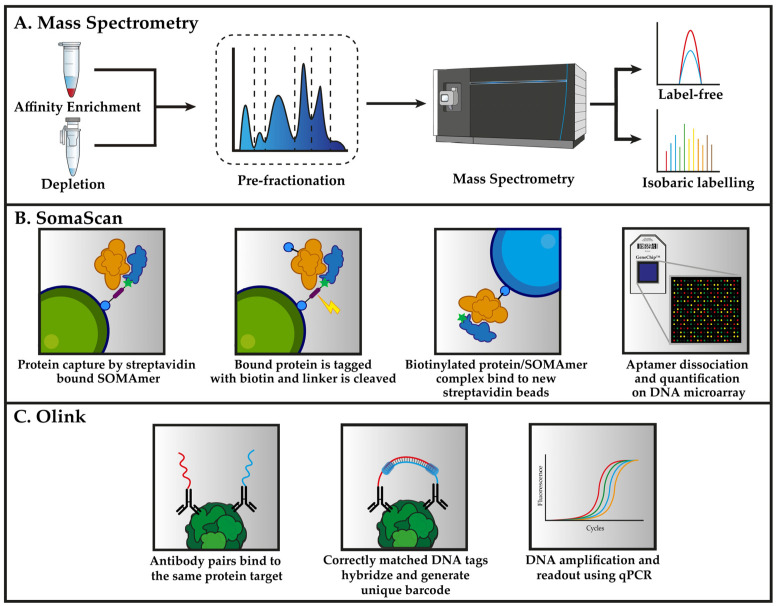
Workflows for the three primary analytical techniques that were deployed for proteomic analysis of plasma. (**A**) Mass spectrometry-based methods for the analysis of plasma typically relies on either depletion or enrichment to detect low-abundant proteins. Pre-fractionation prior to proteomic analysis is an optional step that separates the peptides in one dimension, thereby dividing the sample into a smaller fraction for later analysis. The quantification of proteins for explorative biomarker discovery is performed either label-free or using isobaric labelling reagents. (**B**) The SomaScan assay uses SOMAmers (blue) that are synthesized with a fluorophore, photocleavable linker as well as biotin, which is used to immobilize the SOMAmers to streptavidin beads. SOMAmer are able to capture proteins (orange) from solution followed by biotinylating of the captured proteins. The photocleavable linker is destroyed using external UV light to release the SOMAmer/protein complex back into the solution. Biotin-tagged proteins are recaptured on secondary streptavidin beads. SOMAmer dissociation is caused by denaturing of the captured protein, thereby allowing the SOMAmer reagents to hybridize to complementary sequences on a microarray chip. The abundance of each protein is derived from the amount of fluorescent intensity that is detected from each fluorophore. (**C**) Antibodies with the specificity for the same protein (green) are brought into close enough proximity that the attached DNA can hybridize and extend in the presence of a DNA polymerase. This process forms unique DNA barcodes for each protein in the assay, which can then be amplified and used for quantification using qPCR.

In the remaining sections, we will introduce the most recent and commonly used proteomic methods that are applied to plasma proteomics in cardiovascular medicine. This includes, mass spectrometry, aptamer-based microarray (SomaScan), and immunoaffinity assays (Olink). The principle of these methods are summarized in Figure 1.

### 4.1. Mass Spectrometry-Based Plasma Proteomics

#### 4.1.1. Preanalytical Steps in Mass Spectrometry-Based Plasma Proteomics

As mentioned above, the complexity of the plasma proteome hinders the detection of lower abundant proteins by mass spectrometry-based proteomics. Therefore, much effort has been devoted to the development of methods, such as selective precipitation using acetonitrile [35], protein equalization using combinatorial peptide ligand libraries (ProteoMiner) [36], or immunodepletion for the removal of up to the 50 of the most abundant proteins in human plasma [37,38] prior to mass spectrometry analysis. Currently, the commonly used methods for mass spectrometry-based plasma proteomics are the two latter ones. Despite the fact that immunodepletion methods and the protein equalization methods [37,38] reduce the signals from high abundant proteins in the mass spectrometry analysis, they do not allow the detection of low abundant plasma protein by mass spectrometry. Recently, a method that was based on the affinity capture of low-abundant plasma proteins using small-molecule affinity-based probes was developed [39]. A total of four different small-molecule affinity-based probes that were based on agarose-immobilized p-aminobenzamidine (ABA), 8-amino-hexyl-ATP (ATP), 8-amino-hexyl-cAMP (cAMP), or O-phospho-L-tyrosine (pTYR) were explored for their capability to enrich lower abundant proteins while also removing high-abundant proteins. They demonstrated excellent removal of high abundant plasma proteins and selective enrichment of low abundant plasma proteins as compared with the immuno-depletion and protein equalization and outperformed the other methods. Compared to undepleted plasma, a more than an 80% increase in protein identification was obtained based on these methods [39]. This method was applied for the proteomics detection of low-abundant proteins that were originally identified as biomarkers for AMI, which, for a large part, turned out to be confounded by heparin administration [40], as well as in a study that identified low abundant protein biomarkers for cardiogenic shock [41]. In addition to the challenge of detecting low abundant proteins, the introduction of contaminants during sample handling and the use of detergents is also something to consider during sample preparation as it can negatively influence the quality of the data that are generated [42]. Other issues regarding incomplete proteolysis, inadequate protein solubilization, and lack of a desalting step are important factors that can have an impact on experiments [43].

#### 4.1.2. MS-Based Proteomics: LC-MSMS Analysis

The preferred method for high throughput proteomic analysis of human plasma has, since the early 2000s, been liquid chromatography combined with mass spectrometry (LC-MS) [44,45]. Generically, a mass spectrometer measures mass–charge ratios of ionized analytes (e.g., peptides or proteins) within a gas phase. Although many diverse types of mass spectrometers exist, the workflows for plasma proteomics are typically based on the depletion of high-abundant proteins or enrichment of low abundant proteins. This occurs via the proteolytic cleavage of the proteins into well-defined peptides by restriction enzymes, typically trypsin, followed by chromatographic separation using nano-flow LC and mass spectrometric detection that measures both the accurate mass and the amino acid sequence of the tryptic peptides. The main issue with LC-MS applied to plasma proteome analysis is that it is not possible with this method to measure across the entire dynamic range of plasma, and it, therefore, requires additional analytical steps [10]. The large majority of mass spectrometry-based analyses on plasma is performed with what is known as a bottom-up proteomic approach [46]. In bottom-up proteomics (sometime referred to as shotgun proteomics), the proteins are proteolytically digested and are then chromatographically separated (in one or several dimensions) and the analysis is performed on the resulting peptides. In principle, two complementary approaches are employed for peptide measurements using MS; untargeted MS and targeted MS.

For un-targeted MS, two methods for data-acquisition are applied for MS-based plasma proteomics: data-dependent acquisition (DDA) and data-independent acquisition (DIA). The most commonly used mode of data-acquisition in mass spectrometry is DDA in which precursor ions whose intensity exceed a pre-defined threshold in a survey scan (MS1) are automatically selected for fragmentation. This (semi) stochastic way of selecting precursor ions is inherently biased toward the most abundant ions and also leads to the selection of different subsets of ions in subsequent analytical runs leading to a higher degree of missing values and less reproducibility between runs [47]. Not surprisingly, this also implies that the biologically relevant precursor ions that fail to meet the pre-defined threshold or are co-eluted with more abundant ions will not necessarily be selected for fragmentation [48]. A way to reduce the influence of high abundant ions is the use of dynamic exclusion which prevents the repeated acquisition of precursor ions and can have a noticeable effect on the number of the identified proteins. The downside of bottom-up proteomics is that the identification of proteins is limited to the canonical amino acid sequences and, less reliably, the isoforms of said canonical proteins, unless their amino acid sequences are present in the database that is being used for searching the data. The identification of different proteoforms is hindered largely due to the proteolytic digestion which is an unavoidable aspect of bottom-up proteomics. On the other hand, the nature of PTM can be localized by DDA acquisition, thereby enabling the investigation of the relevance of PTMs in cardiovascular diseases [49] as long as the PTM is located in a canonical protein sequence on sequencing that is present in the database that is being applied for searching the raw data. The processing of raw data from DDA have been made easily available with both commercial and open-source pipelines for the identification of protein identities [50,51,52]. In DDA analysis, protein identities are inferred based on sequencing of the proteolytically digested peptide by searching the derived peptide sequence against a protein sequence database, such as UniProt [53].

With DIA, some of the shortcomings of DDA, such as stochastic and irreproducible ion selection, is alleviated by removing the direct dependency on ion intensity for ion fragmentation. A typical implementation of DIA is sequential window acquisition of all theoretical mass spectra (SWATH-MS). In its first implementation by Gillet et al. [54], a quadrupole-Time-of-Flight (q-TOF) mass spectrometer was used to cycle through 32 consecutive, overlapping (1 *m*/*z*) precursor isolation windows with a width of 25 Da (so called swaths) for systematic and unbiased fragmentation of precursor ions that fell within the predetermined isolation windows. While data acquisition with DIA is fairly simple, the analysis of DIA data is still currently a challenge due to the multiplexed MS2 spectra that are generated due to the co-fragmentation of co-eluting precursor ions. Originally, DIA data was analyzed using similar database search engines that were used in DDA data analysis. However, Gillet et al. also devised a novel data analysis workflow in which prior knowledge of previously observed peptides that were contained in spectral libraries was used to search the generated data. Generally, DIA has several advantages in relation to DDA analysis, such as higher reproducibility and retrospective querying [55,56].

With targeted MS, stable isotope peptides are used as reference points to provide absolute quantification of the peptides that are present in the sample. Targeted MS, also referred to as multiple reaction monitoring (MRM) is predominantly used for studies where a relatively small number of preselected proteins are measured. For example, for the validation of protein biomarkers in large sample cohort that was independent of the cohort that was analyzed, a more global untargeted proteomic strategy was used to initially discover them. While MRM is usually performed with triple-quadrupole instruments, another implementation is parallel reaction monitoring (PRM) in which the high-resolution Orbitrap or alternatively a time-of-flight (TOF) is used as an MS2 analyzer [57,58]. The drawback of targeted MRM analysis is the need for prior knowledge of the peptides of interest with regards to the transitions of the fragment ions [59]. This drawback is, however, outweighed by the fact that MRM provides a more sensitive and also an absolute quantification which enables a comparison of results across studies from different laboratories. This limitation is alleviated with PRM in which prior knowledge of the target transitions is unneeded due to the simultaneous monitoring of product ions of a targeted peptide with high resolution. Both SRM and PRM have demonstrated comparable sensitivities, repeatability, and dynamic range for targeted quantification [60,61]. A classic example in cardiovascular research where protein biomarkers are initially identified by untargeted MS and validated by targeted MS include the identification and validation of vinculin as plasma biomarker for acute coronary syndrome [62].

#### 4.1.3. Quantitative MS-Based Proteomics

One of the key important prerequisites for success in finding new, CVD-specific biomarkers are that the methods that are used are quantitative, that these methods have high analytical precision, and that there is a sufficient number of subjects in the cohort being studied, i.e., that the study is adequately powered. The quantification of proteins in DDA discovery studies is performed in two ways: as label-free quantification, or with the use of protein-labelling reagents, so-called isobaric tags. Label-free quantification extracts the MS1 peptide precursor ion chromatogram and uses either the intensity of the highest point or integrates the peak area over the chromatographic time scale [63] as a quantitative measure. Another way of label-free-quantification is spectral counting in which the number peptide spectrum matches (PSMs) from identified unique peptides from a given protein are summed as the number of PSMs that have been shown to correlate with the protein quantity [64]. Label-free quantification methods often leads to a higher degree of missing values in the quantification step due to the stochastic sampling characteristic of DDA analyses meaning that the same proteins are not identified and quantified across different samples [65]. A process that is primarily known as match-between runs (available in free-to-use software, such as MaxQuant) in which protein identities and quantification can be transferred between files based on MS1 scans, leads to significant reduction in the number of missing values [66].

With quantification using isobaric tags, isobaric amine-reactive tags are covalently coupled to the reactive amino groups of lysine and the peptide N-terminus [67]. Different tagging methods are commercially available [68,69] and allows the multiplexing of up to 18 and subsequently analyze them in one single MS experiment [70]. This analytical setup is increasingly being applied to large-scale proteomics studies allowing for highly complex study designs [71,72] for the analysis of a large number of patient samples. In a multiplex isobaric-tagging experiment, each individual sample is digested and peptides are labeled/tagged with a unique label/tag and pooled in equimolar proportions. The peptides tagged with labeling reagent are fragmented with high-energy collision-induced dissociation (HCD) during analysis and the mass reporter ions are released [73]. The intensities of each different mass reporter ion are then representative for the relative concentration of the measured peptides in each sample. The use of isobaric-tagging enables high-throughput analysis which reduces the instrument time, variation between runs, and the extent of missing values [74].

### 4.2. Affinity-Based Proteomics Methods

The pace by which new developments of affinity-based techniques that are used in sample processing for increasing the measurable dynamic range, have increased rapidly. Typically, depletion methods as well as substantial fractionation have been employed to lessen the effect of high abundant proteins in mass spectrometry analysis.

In recent years, several commercially available affinity-based platforms promising high-throughput analysis of plasma with high sensitivity and the capacity to analyze several hundred to thousands of proteins in a multiplex manner and have been developed and applied in numerous CVD biomarker discovery studies. The plasma proteome has an enormous dynamic range and variability, including splice variants, cleavage products, and posttranslational modifications. Antibody-based techniques have, so far, predominated, but recent developments in affinity-based techniques such as the SomaScan aptamer assays (SomaLogic), proximity extension assays (Olink), and microbead-based multiplex immunoassay (xMAP), also promise the multiplexed measurement of proteins in a scalable manner.

#### 4.2.1. Aptamer Microarrays (SomaScan)

Methods that use DNA or RNA scaffolds as binding reagents have emerged during recent years and one of these is the SomaScan assay, which uses single-stranded DNA-based protein affinity reagents, termed SOMAmer (Slow Off-rate Modified Aptamer). Publications which use the SomaScan-platform have increased over the years, particularly in the search for novel CVD plasma biomarkers. The attraction is easily understood as each iteration of the platform has increased the number of proteins that can be measured in a single run, with the latest iteration of the platform offering ≈ 7000 protein measurements. The platform utilizes aptamers (short oligonucleotides) with binding affinities to the epitopes of the selected proteins, which are multiplexed allowing the simultaneous quantification of multiple proteins at the same time. The SomaScan assay exploits the versatility of oligonucleotides in their capability of binding specific proteins as well as being detectable by DNA detection methods. Modified nucleotides that are combined with an artificial iterative selection process of appropriate aptamers (SELEX) enables the creation of reagents with specificity for target proteins [75]. Overall, the SomaScan assay targets a large subset of the human plasma proteome but claims to overcome classical dynamic range limitations that are observed with MS methods. While the SomaScan assay is best known for high throughput screening of clinical samples in typical biofluids, e.g., serum, plasma, and cerebrospinal fluid, others have also used the assay to study exosomes and cell extracts [76,77,78,79]. Moreover, SomaScan assay was recently applied to the proteomic analysis of plasma in relation to heart failure [80] and acute myocardial infarction (AMI) [81].

#### 4.2.2. Proximity Extension Assays (Olink)

Another recent development in multiplex affinity-based plasma proteomics is the proximity extension assay (PEA) technology that was commercialized by Olink Proteomics AB (Uppsala, Sweden). Both exploratory 384-plex kits that were based on a next-generation sequencing (NGS) platform or more targeted solutions using 48-96-plex kits that were based on quantitative real-time PCR (qPCR) platform are available with focus on different groups of proteins (e.g., inflammatory- or cardiovascular-related proteins). The Olink assay uses pairs of antibodies for the dual recognition of target proteins, thereby increasing specificity. Unique DNA oligonucleotides are attached to each antibody and will hybridize only when in close proximity to a matched antibody. The DNA-polymerase-mediated extension of the hybridized oligonucleotides forms a unique barcode-sequence that is specific for each protein. The created sequence can then be quantified using either qPCR or NGS (depending on the kits used) as the initial concentration of target proteins is proportional to the quantity of generated sequences through the polymerase reaction. The Olink platform has recently been applied for investigating circulating biomarkers for heart failure [82] and coronary heart disease [83].

#### 4.2.3. Microbead-Based Multiplex Immunoassay (xMAP)

The Luminex xMAP (Multi-Analyte profiling) technology uses different sets of microspheres in either a magnetic or non-magnetic bead format. Microsphere sets are internally dyed with two spectrally different fluorophores in different concentrations which creates unique spectral signatures for each microsphere set. Each microsphere set can be coated with various capture molecules, facilitating the capture of up to 100 analytes at the same time. Excitation with a dual laser system enables differentiating between the different microsphere sets and parallel measurement of the fluorescent reporter molecule, which has been captured with the assay. The xMAP platform has recently been used to investigate biomarkers for different cardiovascular diseases [84] and another study investigating atrial fibrillation [85].

## 5. Strengths and Limitations of the Current, Major Plasma Proteomics Technologies

Since mass spectrometry is an older method of analyzing proteins, it also means that the weaknesses and limitations of using mass spectrometry are more thoroughly elucidated than new emerging methods. The challenges of high complexity samples with wide dynamic ranges, such as plasma, are well described and sample preparation techniques as well as instrument methods for handling those challenges are continuously being developed and improved upon. Nonetheless, the detection and quantification of low-abundant proteins is difficult.

The various technologies that are presented herein are vastly different in their implementation as well as their strengths and limitations, which can influence the important decision of which technology to make use of for analyzing the plasma samples in precious clinical cohorts. While no direct systematic comparison between the three technologies has been carried out, several aspects have been investigated regarding proteome coverage, specificity, and other aspects that are relevant for plasma proteomics studies (Table 2).

**Figure 2:**
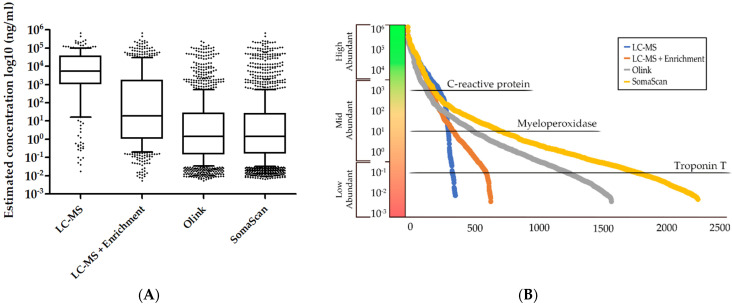
The estimated plasma protein concentrations from the publicly available Peptide Atlas build (Human Plasma 2021-07, accessed on 1 December 2021) [24] was used to compare the number of quantifiable protein from LC-MS analysis (with and without affinity enrichment) with proteins that were available in the Olink and SomaScan assays. Note: Not all the proteins were associated with an estimated plasma protein concentration in the Peptide Atlas build. (**A**) Boxplot of the estimated protein concentrations for each of the analytical techniques displaying the distribution of quantifiable proteins. The whisker boundary is defined as the 10th–90th percentile. (**B**) Graphical representation of the diversity in the number of quantifiable proteins in relation to the estimated protein concentrations with known cardiovascular biomarkers as examples of the dynamic range in plasma. While the curves are initially similar, it is evident that the SomaScan and Olink are able to quantify far more mid-and low-abundant proteins compared to LC-MS analysis even with the application of affinity enrichment. LC-MS analysis using affinity enrichment is able to sporadically quantify proteins in the same lower range as SomaScan and Olink. Dataset references: LC-MS: [39]; LC-MS + Enrichment: [40]; Olink: [89]; SomaScan: [90].

MS-based analysis of plasma benefits from the long history of usage within the field of proteomics and the continued acceleration of advancements within instrument method development that is carried forward by both manufacturers and researchers alike. Researchers utilizing MS-based methods for analysis of plasma also benefit from the vast existing resources that are available with regards to protocols, software, and open repositories that can support the development or refinement of methods for faster and more sensitive analyses. As previously mentioned, the different types of MS-based quantification that is used will have an impact on the completeness of the dataset with label-free quantification, having a higher degree of missing values compared to isobaric labelling, which, in return, will result in fewer protein identifications and a higher cost-per-sample, due to the cost of labelling reagents. Nonetheless, the detection and quantification of low-abundant proteins remains a challenge with MS-based methods, more so than aptamer-or antibody-based methods, which are not affected by the extensive plasma protein concentration range to the same degree (Figure 2).

Major weaknesses of the immunoaffinity-based proteomics technologies are insufficient antibody specificity and antibody cross-reactivity, which may limit the degree of multiplexing that is feasible [86]. This limitation is lessened by the Olink platform, which uses complementary DNA oligonucleotides that are attached to the antibody pairs to decrease this nonspecific cross-reactivity. Another major weakness with immunoaffinity-based technologies is the pre-selection of protein targets which again limits the exploratory use of these technologies. By contrast, the untargeted nature of mass spectrometry-based proteomics is well-suited for exploratory proteomic analysis as no pre-selection of proteins is done by using this technology. Proteomic analysis of CVD animal models have traditionally been performed using mass spectrometry for the discovery of potential biomarkers, but the recent applications of both the Olink and SomaScan assay demonstrate the feasibility of discovering relevant plasma biomarkers for CVD not only in humans but in mouse models [87,88].

The emerging trend of high-throughput affinity-based methods for plasma proteomics have increased the number of proteins that can be quantified into the thousands by taking advantage of the signal amplification of qPCR and DNA microarrays, which easily surpass the plasma proteome coverage that is achievable with mass spectrometry. The analytical speed of mass spectrometry is the major limitation of high-throughput and is largely hindered by the chromatographic separation of peptides which is time-consuming, which aptamer- and antibody-based assays does not suffer from [91]. However, recent innovations in chromatographic and mass spectrometric instrumentation and development of new protocols have increased the analytical speed of mass spectrometry and have enabled larger quantities of samples to be analyzed faster [92,93,94,95]. While affinity-based methods have been used in multiple publications, several studies have reported issues with affinity-binding to non-target proteins. This was exemplified by Sun et al. who demonstrated that 14% of 920 tested SOMAmers displayed cross-reactivity with homologous proteins (>40% sequence homology) with a similar binding affinity [96]. Another study by Williams et al. found that 27% of the reagents that were tested showed affinity for non-target proteins, of which 13% of the reagents displayed similar affinities to the non-target proteins as the target proteins [90]. They found (similar to Sun et al.) that the non-target proteins which were wrongfully bound by the reagents were highly similar to the target proteins. Another recent study investigated, among other things, the correlation between Olink and the SomaScan assay and found that the range of correlations coefficients varied greatly among the 425 proteins that were compared (Spearman’s rho from −0.58 to 0.93) [97]. Cross-reactivity of aptamers and antibodies was hypothesized to be a factor in the low correlation between assays for proteomic analysis. It has also been noted for the aptamer-based assays that the type of anti-coagulant that is used can influence the degree of variation that is observed. Kim et al. found that the proportion of CV’s that were lower than 10% was higher for proteins that were measured in heparin plasma (92%) than for the commonly used EDTA plasma (66%) [98]. This is a key aspect in planning which proteomic technology to apply in clinical proteomic studies as biobanks usually only collect blood samples in vials employing one type of anti-coagulant and not both. Generally, mass spectrometry does not suffer with low specificity issues due to the nature of mass spectrometry tandem acquisition, which ensures high specificity of the identified proteins. Ongoing advancements within optimized probe design and quality control will likely lead to improvements of the current emerging proteomic assays.

## 6. Recent Developments in Plasma Proteomics of Cardiovascular Diseases

Except in recent years, the majority of the published applications of plasma proteomics for research into cardiovascular diseases has primarily been performed with mass spectrometry-based proteomics in human tissues and animal models. During recent years, however, multiple studies with large population cohorts have been investigated with the use of both mass spectrometry-based and emerging commercially available affinity-proteomics technologies that are based on either aptamer microarrays (SomaScan), multiplex proximity extension assays (Olink) or microbead-based multiplexed immunoassays (Luminex) to profile plasma proteomes. Here, we highlight the most recent and relevant applications of these proteomics platforms in CVD research.

For myocardial infarction, a recent study by Mohammad et al. [99] searched for biomarkers that were related to infarct size and left ventricular ejection fraction (LVEF) in 119 patients from a randomized clinical trial with recent ST-elevation myocardial infarction (STEMI) using multiplex proximity extension assay (Olink). The patients underwent percutaneous coronary intervention and blood samples were obtained at baseline and after 6, 24, and 96 h. The protein profile of 131 cardiovascular and inflammatory-related proteins was correlated with infarct size and LVEF was measured by magnetic resonance imaging. A total of five proteins were shown to be associated with increased infarct size and worse LVEF (ST2, interleukin-6, pentraxin-3, interleukin-10, renin, and myoglobin) and four proteins showed an inverse relationship with respect to infarct size and LVEF (TNF-related apoptosis inducing ligand, TNF-related activation induced cytokine, interleukin-16, and cystatin B). Several proteins were associated with MRI-measured infarct size and LVEF post-STEMI. In another study, the Olink^®^ CARDIOVASCULAR III proximity extension assay was used to investigate a wide range of cardiovascular protein biomarkers in the acute phase of STEMI compared with the stable phase three months after STEMI [100]. A total of 48 STEMI patients were treated with primary percutaneous coronary intervention (PPCI), and blood samples were obtained immediately prior to PPCI and again three months later. Of the 92 proteins that were related to immune and inflammatory response, cell adhesion, and hemostasis, 29 proteins differed significantly even after Bonferroni correction when comparing the acute phase of STEMI with the stable phase three month later. A large portion of these proteins, including insulin growth factor binding proteins, myeloperoxidase, and spondin-1 were found to be confounded by heparin that was administrated immediately prior to blood sample drawing in another study, thereby demonstrating the importance of the thorough consideration of medications prior to blood sampling in CVD biomarker research [40]. It is believed that negatively charged heparin displaces these proteins from the endothelial surface by the binding to the positively charged endothelial surface, thereby dramatically increasing their concentration in the blood [40,101].

Abdominal aortic aneurysm is a common and progressive life-threating disease among the elderly in many countries and with increasing mortality [102]. An effective method to reduce this high mortality is the early detection of the AAA by screening with ultrasound followed by elective surgery. Various challenges that are associated with the establishment of scanning-based screening programs have forced the development of a blood test for AAA prevalence as a cost-effective and minimally invasive way to screen high-risk populations and to determine which patients should receive further diagnostic imaging. This was recently addressed in a study by Hendrikson et al. that investigated plasma samples from 20 patients by mass spectrometry-based proteomics aimed at the identification of potential protein biomarkers that are related to abdominal aortic aneurysm [103]. The plasma samples from 12 patients and 8 controls were depleted for high abundant plasma proteins such as albumin and immunoglobulins, tagged with isobaric tags, and analyzed by two-dimensional liquid chromatography combined with mass spectrometry. This study identified eight potential biomarkers for AAA, whereof only one (bleomycin hydrolase, BH) was validated by an enzyme-linked immunosorbent assay (ELISA) in an independent cohort of 41 controls and 38 patients with small AAA, 40 patients with large AAA, and 55 patients with previous ruptured AAA. Although significantly regulated in AAA patients, the association of BH and AAA was too weak for BH to be clinically useful as AAA biomarker. A similar MS-based proteomic approach for the identification of plasma biomarkers for AAA was used by Burillo et al. [104], and this study found that apolipoprotein A1 (ApoA1) was negatively correlated with AAA size and thrombus volume, but positively correlated with HDL-Cholesterol levels in plasma and the authors claimed that targeting HDL functionality may halt AAA development. A range of other circulating protein biomarkers for AAA have been investigated. None of them have, however, been shown to be useful in diagnosing and predicting AAA growth [105], and new proteomics studies in larger cohorts are, therefore, needed to find clinically useful AAA plasma biomarkers for screening and stratification purposes.

Since its appearance, the SomaScan platform has been used in numerous proteomics CVD studies including heart failure, AMI, planned myocardial infarction (PMF), and also in combination with genetic tools to identify novel genetic determinants of proteins that are associated with the risk of developing a cardiovascular disease. In an early study, the aptamer assay was applied to planned myocardial infarction measuring 1129 plasma proteins [106] that later was scaled to 4783 plasma proteins [107] in a relatively small cohort, demonstrating the power and potential of this parallelized proteomics platform. Another recent application of SomaScan was the analysis of plasma from two separate cohorts of patients with a previous incidence of AMI where a subset of these patients subsequently experienced heart failure (181 patients in the first cohort; 33 cases in the second cohort). The study found several plasma proteins that were related to post-AMI heart failure, including the well-described biomarkers NTproBNP and troponin T, as well angiopoietin-2, thrombospondin-2, latent transforming growth factor-β binding protein-4, and follistatin-related protein-3 [81]. In the pioneering study by Benson et al. [108] the SomaScan assay was applied to study the association of genetic variants with the plasma concentrations of a panel of previously identified Framingham risk score-associated plasma proteins using linear mixed effects models in two population-based cohorts. SomaScan discovery analyses measuring 1129 plasma proteins was done in 759 individuals of the Framingham Heart Study (FHS) offspring cohort, and validation was done in a cohort of 1421 individuals from the Malmö Diet and Cancer Study. This study identified several novel genetic determinants of proteins that were associated with the FRS including the novel genetic association with plasma levels of apolipoprotein E. More recently, the aptamer platform was applied for the proteomic analysis of 1017 patients that were diagnosed with heart failure with concurrent reduced ejection fraction (HFrEF) for the prediction of risk of death or deteriorating heart failure [109]. The researchers analyzed 4453 slow off-rate modified aptamers in the SomaScan and identified eight proteins that were associated with different cardiovascular and heart failure phenotypes. A protein risk score was developed and used in combination with a validated clinical risk score (MAGGIC) and NT-proBNP. The risk score improved risk stratification of HFrEF patients moderately when compared to the combination of MAGGIC and NT-proBNP only. As noted by the authors, the study lacks proper validation by an alternative method which would have been appreciated given the known limitations of the SomaScan.

The Luminex xMAP platform was recently deployed in an explorative study, in which several biomarkers that were related to a variety of cardiovascular diseases were found to have markedly different expression between the sexes, demonstrating the complexity of clinical diseases such as cardiovascular diseases [84]. A total of 71 circulating biomarkers that were specific for CVDs were measured in 7184 participants from the Framingham Heart Study, of which 61 biomarkers displayed significant differences between the sexes. Most notably, the study found several biomarkers for CVDs that were only associated with CVDs in one of the sexes, for example apolipoprotein B-100 was only associated with incident heart failure in women, but not in men. Likewise, pro-basic platelet protein was only associated with lower risk of cardiovascular death in women compared to men. Another study investigated 85 plasma proteins for their possible relationship to atrial fibrillation in 3378 participants without atrial fibrillation from the Framingham Heart Study, using the Luminex xMAP platform [110]. Of the 3378 participants, 401 participants experienced atrial fibrillation during follow-up. The researchers found that increased levels of IGFBP1 and NT-proBNP as well as decreased levels of IGF1 was associated with a higher risk of incident atrial fibrillation.

Additionally, plasma samples from 299 patients were examined with mass spectrometry with the goal of discovering biomarkers that were related to the severity of coronary artery atherosclerosis [111]. The researchers performed initial discoveries in 18 plasma samples, which was depleted for 14 high abundant proteins using immunodepletion (MARS14) and was then labelled with TMT 6-plex and fractionated. Conventional LC-MS/MS analysis was then employed to analyze the discovery cohort and selected 52 differentially expressed candidate biomarkers for further investigation. The candidate biomarkers from the smaller discovery cohort were then quantified in a larger verification cohort consisting of 53 patients. The plasma samples were similarly subjected to immunodepletion and analyzed using MRM for verification. Finally, seven candidate biomarkers were validated in 228 patients, where the plasma samples were also immunodepleted prior to analysis with MRM for validation of the discovered markers. Of the seven biomarkers, four were found to be up-regulated and one protein to be down-regulated in diabetes patients with severe coronary artery stenosis. The remaining two proteins were downregulated in patients without diabetes with severe coronary artery stenosis.

## 7. Conclusive Remarks and Outlook

Ideally, technologies that are adopted for the discovery of novel CVD plasma protein biomarkers should have the capability of robustly measuring virtually all proteoforms of proteins that are present in a plasma sample in a parallelized manner irrespective of the protein dynamic concentration range. No such technology exists. Where mass spectrometry-based proteomics has the capability of measuring various proteoforms resulting from post-translational modifications or genetic variations, even in a quantitative manner this technology is significantly challenged by the dynamic concentration range of plasma proteins as the considerable difference in protein concentrations surpasses the dynamic measuring range of MS instruments by several orders of magnitude. However, this drawback has been addressed by the recent development of sample preparation methods, which either remove the high-abundant plasma proteins or enrich the low-abundant plasma proteins prior to mass spectrometric analysis. By contrast, affinity-based methods such as SomaScan or Olink measure plasma proteins across the entire concentration range of plasma proteins but may be challenged by lower specificity for the targets. Moreover, mass spectrometry-based proteomics methods also struggle with a medium–low sample throughput and is currently outcompeted by the affinity-based assays that analyses up to hundreds of samples per day. We envision that future efforts in technological development will focus on increasing the specificity and also the numbers of the aptamers that are utilized in assays to increase the validity of measurements and the number of proteins that are measured. The continuous development of antibody production will likely increase the number of proteins that can confidently be quantified using the antibody-based assays that were mentioned herein. For MS-based proteomics, future improvements of sample preparation and multiplex capacity of the TMT reagents combined with better instruments offering increased sensitivity and scan rate will benefit from LC-MS-based clinical plasma proteomics. Moreover, further development of novel data-acquisition methods combined with increasing use of automation in sample preparation will further strengthen the LC-MS technology as a whole and will likely enable the analysis of a higher number of proteins in more patients at a significant increased throughput. These future technological developments within proteomics will, overall, benefit the discovery of new, clinically useful biomarkers in cardiovascular medicine.

## Figures and Tables

**Table 1:** Pre-analytical variables that could lead to poor blood sample quality.

Common Errors Related to Venous Blood Collection
Mislabeling
Wrong collection tube
Insufficient volume drawn
Sample contamination
Incorrect sample handling
Hemolysis
Prolonged storage at incorrect temperature

**Table 2:** Comparison of the current, major plasma proteomics technologies.

	MS-Based	Aptamer-Based	Immunoaffinity-Based
Types	Discovery (DDA) Targeted (DIA, SRM/MRM)	Nucleic acid-binders (aptamers)	Antibody-dependent
Quantification	Relative: Label-free, isobaric labelling Absolute: targeted stable isotope standards	Relative only	Relative (Olink) or absolute
Proteome coverage in plasma	Method/instrument dependent, 250 to 2000	≥7000	Depends on the kits used, 1 to 384 (Olink)
Sample throughput	Protocol-dependent, high throughput is achievable	High throughput	High throughput
Reproducibility	Low intra-assay CV with isobaric labelling or targeted. Generally, higher with label free	Low intra-assay CV	Low intra-assay CV
Specificity	High	Modest to high, known issues with unspecific binding and cross-reactivity	Modest to high when using antibody pairs (Olink)
Identification of different proteoforms (PTMs, isoforms, etc.)	Easy. Existing workflows available	Challenging. Requires development of specific aptamers	Challenging. Requires specific antibodies
Availability/Expertise required	Mostly in-house, which requires high level of expertise. Commercial solutions exist.	Commercial—no expertise required	Commercial or in-house

DDA: data-dependent acquisition; DIA: data-independent acquisition; SRM: single reaction monitoring; MRM: multiple reaction monitoring; CV: coefficient of variation.

## Data Availability

This review article referenced all the articles that were discussed. There are no further data to report.
